# Deregulated expression of Nucleophosmin 1 in gastric cancer and its clinicopathological implications

**DOI:** 10.1186/1471-230X-14-9

**Published:** 2014-01-10

**Authors:** Mariana Ferreira Leal, Tatiane Katsue Furuya Mazzotti, Danielle Queiroz Calcagno, Priscila Daniele Ramos Cirilo, Margarita Cortes Martinez, Samia Demachki, Paulo Pimentel Assumpção, Roger Chammas, Rommel Rodríguez Burbano, Marília Cardoso Smith

**Affiliations:** 1 Genetics Division, Department of Morphology and Genetic, Federal University of São Paulo, R. Botucatu, 740, São Paulo, SP CEP 04023-900, Brazil; 2 Department of Orthopedics and Traumatology, Federal University of São Paulo, São Paulo, SP 04038-031, Brazil; 3 Experimental Oncology Laboratory, Department of Radiology, School of Medicine, University of São Paulo, São Paulo, SP 01246-903, Brazil; 4 Center for Translational Oncology, São Paulo State Cancer Institute, São Paulo, SP 01246-000, Brazil; 5 Research Center of Oncology, João de Barros Barreto University Hospital, Federal University of Pará, Belém, PA 60673-000, Brazil; 6 Human Cytogenetics Laboratory, Institute of Biological Sciences, Federal University of Pará, Belém, PA 66073-000, Brazil

**Keywords:** Gastric cancer, Nucleophosmin, Gene expression, Protein expression

## Abstract

**Background:**

The process of gastric carcinogenesis still remains to be elucidated. The identification of genes related to this process may help to reduce mortality rates through early diagnosis and the development of new anticancer therapies. Nucleophosmin 1 (NPM1) acts in ribosome biogenesis, centrosome duplication, maintenance of genomic stability, and embryonic development. Recently, NPM1 has been implicated in the tumorigenesis processes. Here, we evaluated NPM1 gene and protein expression in gastric tumors and in corresponding non-neoplastic gastric samples.

**Methods:**

NPM1 protein expression was determined by Western blot in 17 pairs of gastric tumors and corresponding non-neoplastic gastric tissue. The protein immunoreactivity was observed in 12 tumor samples. mRNA expression was evaluated by reverse transcription quantitative polymerase chain reaction (RT-qPCR) in 22 pairs of gastric tumors and in matched non-neoplastic gastric tissue.

**Results:**

NPM1 protein expression was significantly reduced in gastric cancer samples compared to matched non-neoplastic gastric samples (*P* = 0.019). The protein level of NPM1 was reduced at least 1.5-fold in 35% of tumors compared to paired non-neoplastic gastric tissue. However, NPM1 immunoreactivity was detected in neoplastic and non-neoplastic cells, including in intestinal metaplastic, gastritis and inflammatory cells. NPM1 was mainly expressed in nucleus and nucleolus subcellular compartments. The staining intensity and the percentage of immunoreactive cells varied among the studied cases. The *NPM1* mRNA level was reduced at least 1.5-fold in 45.5% of samples and increased in 27.3% of samples. An inverse correlation between protein and mRNA expression was detected (r = -0.509, *P* = 0.037). Intestinal-type gastric cancer presented higher mRNA levels than diffuse-type (*P* = 0.026). However, reduced NPM1 protein expression was associated with intestinal-type gastric cancer compared to matched non-neoplastic gastric samples (*P* = 0.018). In addition, tumors from patients with known distant metastasis presented reduced NPM1 protein levels compared to tumors from patients without distant metastasis (*P* < 0.001).

**Conclusion:**

Although the expression of NPM1 is heterogeneous in gastric tumors, our results suggest that NPM1 down-regulation may have a role in gastric carcinogenesis and may help in the selection of anticancer treatment strategies.

## Background

Gastric cancer (GC) is the fourth most common cancer and the second leading cause of cancer-related death worldwide [1]. Although this neoplasia is a serious public health problem due to its high incidence and mortality, little is known about the molecular events involved in gastric carcinogenesis.

Our group recently performed a proteomic analysis aiming to identify proteins with a role in gastric carcinogenesis [2]. In this study, we observed reduced expression of nucleophosmin 1 (NPM1, also known as B23, numatrin and NO38) in several gastric tumors compared to non-neoplastic gastric samples by two-dimensional electrophoresis and mass spectrometry. NPM1 is a nucleolar phosphoprotein that shuttles continuously between the nucleus and the cytoplasm (see review [3]). NPM1 function is not completely known. NPM1 is a member of the nucleoplasmin family of histone chaperones that favor DNA-histone and nucleosome assembly in vitro and also interact with a wide range of unfolded proteins, inducing proper folding in the active state [4]. These multifunctional proteins act in ribosome biogenesis [5], centrosome duplication [6,7], maintenance of genomic stability, and embryonic development [8].

Not surprisingly, NPM1 has been implicated in tumorigenesis processes. NPM1 overexpression was described in solid tumors of diverse histological origins, including astrocytomas [9], as well as colon [10], hepatocellular [11], bladder [12], breast [13], ovarian [14] and prostate [15] carcinomas. Deletions and chromosomal translocations involving the *NPM1* locus (5q35) were described in hematological malignancies (see reviews [16,17]) and lung cancer [18]. Mutations of *NPM1* were also described in hematological malignancies, and it has been suggested that *NPM1*-mutated acute myeloid leukemia is a distinct leukemia entity [19].

NPM1 seems to play a role as both a tumor suppressor and an oncogene. For its tumor suppressor activity, NPM1 seems to act directly and indirectly on the regulation of p53 [3]. On the other hand, NPM1 is also involved in transcriptional activation of some oncogenes, such as *MYC*[20]. Therefore, NPM1 overexpression leads to increased cell growth and proliferation and inhibits differentiation and apoptosis [3].

To our knowledge, only two studies have evaluated *NPM1* mRNA expression in a small set of human primary GC [21,22]. Thus, the role of NPM1 in gastric carcinogenesis remains to be elucidated. In the present study, we analyzed NPM1 mRNA and protein expression in GC and matched non-neoplastic gastric samples. We also evaluated the possible associations between NPM1 and clinicopathological characteristics.

## Methods

### Tissue samples

*NPM1* mRNA expression was evaluated in 22 pairs of GC samples and matched non-neoplastic gastric tissue (>5 cm from the edge of the tumor). In 17 pairs of these GC samples and corresponding non-neoplastic gastric tissue, the protein expression was also evaluated. The protein immunoreactivity was assessed in 12 tumors.

All the gastric samples were obtained from patients who underwent gastrectomy for GC at João de Barros Barreto University Hospital (HUJBB) in the State of Pará, Northern Brazil, during the period from 2006 to 2010. Informed consent with approval of the ethics committee of HUJBB was obtained. All patients had negative histories of exposure to either chemotherapy or radiotherapy before surgery, and there was no co-occurrence of other diagnosed cancers.

Part of the dissected tumor samples was formalin-fixed and paraffin embedded (FFPE). Sections of FFPE tissue were stained with hematoxylin-eosin for histological evaluation or used for immunohistochemistry analysis (IHC). The other part of tumors and the paired non-neoplastic tissue specimens were immediately cut from resected stomachs, frozen in liquid nitrogen and kept at -80°C until protein and nucleic acid extraction.

Table 1 shows the clinicopathological characteristics of the GC samples. All samples were classified according to Laurén [23], and tumors were staged using standard criteria by TNM staging [24]. The presence of *H. pylori*, a class I carcinogen, in GC and non-neoplastic samples was detected by PCR assay. PCR for the urease gene [25] and for the *H. pylori* virulence factor cytotoxin-associated gene A (CagA) [26] was performed as previously reported using the DNA purified simultaneously with the proteins and the mRNA. All reactions were performed in duplicate. In each PCR experiment, positive and negative controls were included. A sample was considered positive if a clear and visible band was observed on the electrophoresis gel (2% agarose gel). In our sample, all GC and non-neoplastic samples presented *H. pylori* infection.

**Table 1:** Clinicopathological characteristics NPM1 expression in gastric cancer samples

	**NPM1 protein**			***NPM1 *** **mRNA**		
**Variable**	**N**	**Ratio T/N (Mean ± SD)**	***P *** **value**	**N**	**RQ (Mean ± SD)**	***P *** **value**
**Gender**						
Male	8	0.81 ± 0.36	0.431	12	2.47 ± 3.86	0.657
Female	9	0.67 ± 0.39		10	1.76 ± 3.43	
**Onset (years)**						
< 45	5	0.73 ± 0.48	0.973	7	0.95 ± 0.89	0.298
≥ 45	12	0.74 ± 0.34		15	2.70 ± 4.25	
**Tumor location**						
Cardia	2	0.87 ± 0.04	0.601	2	0.17 ± 0.19	0.429
Non-cardia	15	0.72 ± 0.39		20	2.34 ± 3.73	
**Histological subtype**						
Diffuse-type	4	0.97 ± 0.25	0.159	6	0.33 ± 0.28	0.026^*^
Intestinal-type	13	0.66 ± 0.38		16	2.83 ± 4.04	
**Differentiation**						
Moderately differentiated	8	0.72 ± 0.38	0.492	10	2.84 ± 4.14	0.990
Poorly differentiated	5	0.57 ± 0.40		6	2.81 ± 4.26	
**Stage**						
Early	4	0.66 ± 0.31	0.675	4	1.02 ± 1.05	0.472
Advanced	13	0.76 ± 0.40		17	2.54 ±4 .01	
**Tumor invasion**						
T1/T2	7	0.78 ± 0.27	0.672	9	1.61 ± 1.63	0.502
T3/T4	10	0.70 ± 0.44		12	2.73 ± 4.67	
**Lymph node metastasis**						
Absent	6	0.78 ± 0.30	0.757	7	1.48 ± 1.82	0.509
Present	11	0.71 ± 0.42		14	2.63 ± 4.31	
**Distant metastasis**						
Unknown/absent	14	0.86 ± 0.26	< 0.001^a^	18	1.21 ± 1.29	0.220
Present	3	0.15 ± 0.20		3	8.49 ± 7.18	

T, tumor sample; N, matched non-neoplastic sample; SD, standard deviation; RQ, relative quantification – gastric tumor samples normalized by matched non-neoplastic sample. ^*^Differentially expressed between groups, *P* <0.05, using t-test for independent samples.

### Protein and mRNA purification

Total protein and mRNA were simultaneously isolated from the gastric tissue samples using the AllPrep DNA/RNA/Protein Kit (Qiagen, Germany) according to the manufacturer's instructions. The protein pellet was dissolved in a buffer containing 7 M urea, 2 M thiourea, 4% 3-[(3-cholamidopropyl) dimethylammonio]-1-propanesulfonate (CHAPS), 50 mM dithiothreitol (DTT), 1% Protease Inhibitor Cocktail (Sigma) and 0.5% each of Phosphatase Inhibitor Cocktails 1 and 2 (Sigma-Aldrich, USA). The protein concentration was determined by the Bradford method (Sigma-Aldrich, USA). The RNA concentration and quality were determined using a NanoDrop spectrophotometer (Kisker, Germany), and the RNA integrity was determined by gel electrophoresis.

### NPM1 protein expression by Western blot

Reduced protein (20 μg) from each sample was separated on a 12.5% homogeneous SDS-PAGE gel and electro-blotted to a polyvinylidene difluoride (PVDF) membrane (Hybond-P, GE Healthcare, USA). The PVDF membrane was blocked with phosphate-buffered saline containing 0.1% Tween 20 and 5% low fat milk and incubated overnight at 4°C with anti-NPM1 (FC-61991, 1:500, Invitrogen, USA) and anti-β-Actin antibodies (ACTB; Ac-74, 1:3000, Sigma-Aldrich, USA). After extensive washing, the PVDF membrane was incubated with a peroxidase-conjugated secondary antibody for 1 hour at room temperature. Immunoreactive bands were visualized using Western blotting Luminol reagent, and the images were acquired using an ImageQuant 350 digital image system (GE Healthcare, Sweden). ImageJ 1.43u software (National Institutes of Health, USA) was used for gel band quantitative densitometric analysis. ACTB was used as a loading reference control. In each experiment, tumor and matched non-neoplastic samples were applied to the same gel. One of the non-neoplastic samples (reference) was applied to all gels to allow comparison among different experiments.

### NPM1 immunoreactivity by IHC

Paraffin sections from 12 different tumor samples were subjected to IHC. Tumor tissue sections (3 or 4 mm-thick) were deparaffinized in xylene and rehydrated in a graded series of ethanol. After heat-induced epitope retrieval, the tissue sections were incubated with primary mouse monoclonal antibody against NPM1 (NA24, 1:200, Neomarkes, USA). A universal peroxidase-conjugated secondary antibody kit (LSAB System, DakoCytomation, USA) was used for the detection system. We used 3.30-diamino-benzidine/H_2_O_2_ (Dakocytomation, Denmark) as the chromogen and hematoxylin as the counterstain. Negative controls in which the primary antibody was replaced by bovine serum albumin (BSA) 5% in phosphate-buffered saline (PBS) were performed in all series, and sections of normal human amygdala tissue were used as positive controls.

The slides were viewed by light microscopy using a Nikon Eclipse E600 microscope (Nikon, NY, USA) equipped with a digital camera Nikon DSM1200F (Nikon, NY, USA). The nonstained region (white region) was selected and set as background. Any staining was considered to be a positive result, irrespective of intensity. An arbitrary semiquantitative score was developed to quantify NPM1 immunoreactivity, as follows: 0, from negative to minimal staining (< 10% of cells); 1+, for those tumors showing a weak staining and over 10% of cells; 2+, for those tumors presenting a moderate staining and over 10% of cells; and 3+, for those tumors presenting a strong staining and over 10% of cells.

### *NPM1* mRNA expression by reverse transcription quantitative polymerase chain reaction (qRT-PCR)

First, complementary DNA was synthesized using the High-Capacity cDNA Archive kit (Applied Biosystems, Poland) according to the manufacturer’s protocol. All real-time RT-qPCR reactions were performed in triplicate for both the target gene (*NPM1*: Hs01576587_g1, Applied Biosystems, USA) and the internal control (*ACTB*: Hs03023943_g1, Applied Biosystems, USA). The relative quantification (RQ) of the gene expression was calculated according to Pfaffl method [27]. A non-neoplastic gastric tissue was designed as a calibrator for all samples for the comparison between neoplastic and non-neoplastic samples. In addition, the non-neoplastic gastric tissue sample was designated as a calibrator for each paired tumor for clinicopathological analysis.

### Statistical analysis

Gene and protein expression data are shown as mean ± standard deviation (SD) for each group. We first evaluated the normal distribution of all data using the Shapiro-Wilk normality test to determine the subsequent use of appropriate tests for statistical comparison. *NPM1* mRNA levels were not normally distributed and were transformed (z-score) for analysis such that they followed a normal distribution. Paired t-tests were performed to compare the mean NPM1 expression between non-neoplastic and tumor samples. The associations between the clinicopathological parameters and the mean NPM1 mRNA and protein expression were assessed using t-tests for independent samples. The possible associations between NPM1 immunoreactivity and clinicopathological parameters were assessed by Fisher’s exact test. The correlation between NPM1 immunoreactivity and mRNA or protein expression by Western blot was analyzed by Spearman’s rank correlation test. The correlation between NPM1 mRNA and protein expression by Western blot was analyzed by Pearson’s test. For the analyses using the t-test for independent samples and the Pearson’s test, the NPM1 expression in tumor samples was calibrated by their matched non-neoplastic counterpart. In all analyses, *P* < 0.05 was considered significant.

## Results

**Figure 1:**
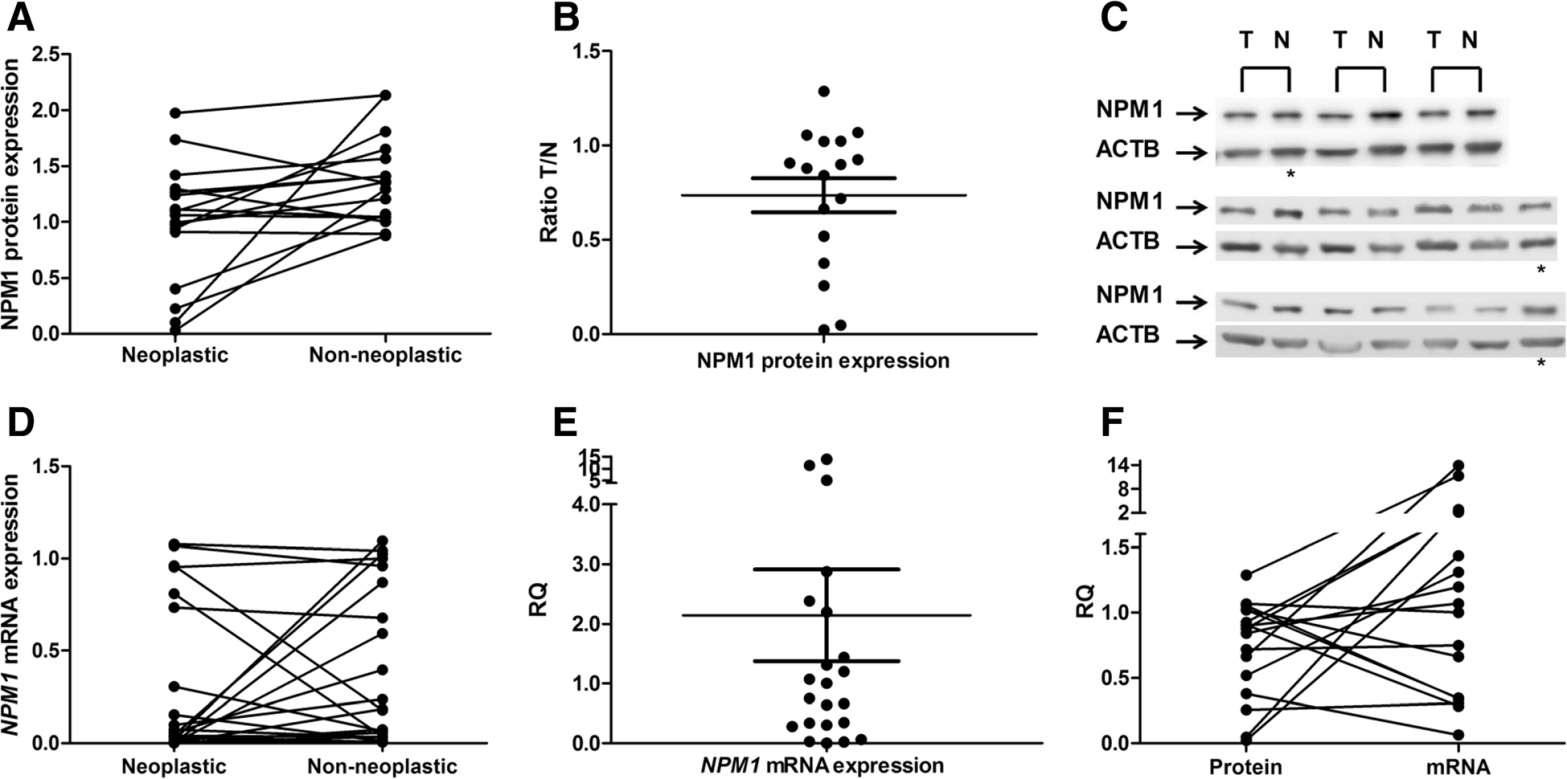
**Expression of NPM1 in gastric samples. (A)** protein expression normalized by ACTB and calibrated by the level of a reference sample; **(B)** the ratio of NPM1 protein expression between tumor and matched non-neoplastic samples; **(C)** Western blot using anti-NPM1 and anti-ACTB antibodies in representative gastric tumors and their matched non-neoplastic counterparts; **(D)** mRNA expression normalized by *ACTB* and calibrated by a non-neoplastic gastric tissue; **(E)** relative quantification of *NPM1* mRNA expression, in which gastric tumors samples were calibrated by the matched non-neoplastic tissue; **(F)** relationship between the relative quantification of NPM1 protein and mRNA levels, in which non-neoplastic samples were used as reference for each matched tumor. T: tumor samples; N: non-neoplastic samples; RQ: Relative quantification. *Reference sample in Western blot gels.

NPM1 protein expression was significantly reduced in GC samples compared to matched non-neoplastic gastric samples (0.99 ± 0.54 *vs* 1.39 ± 0.39, values relative to the reference sample; *P* = 0.019, by paired t-test; Figure 1A). The protein level of NPM1 was reduced at least 1.5-fold (50% decrease in the expression) in 35% of GC samples, and no tumor presented an increase in expression of 50% compared to their paired non-neoplastic gastric tissue (Figure 1B and 1C).

**Figure 2:**
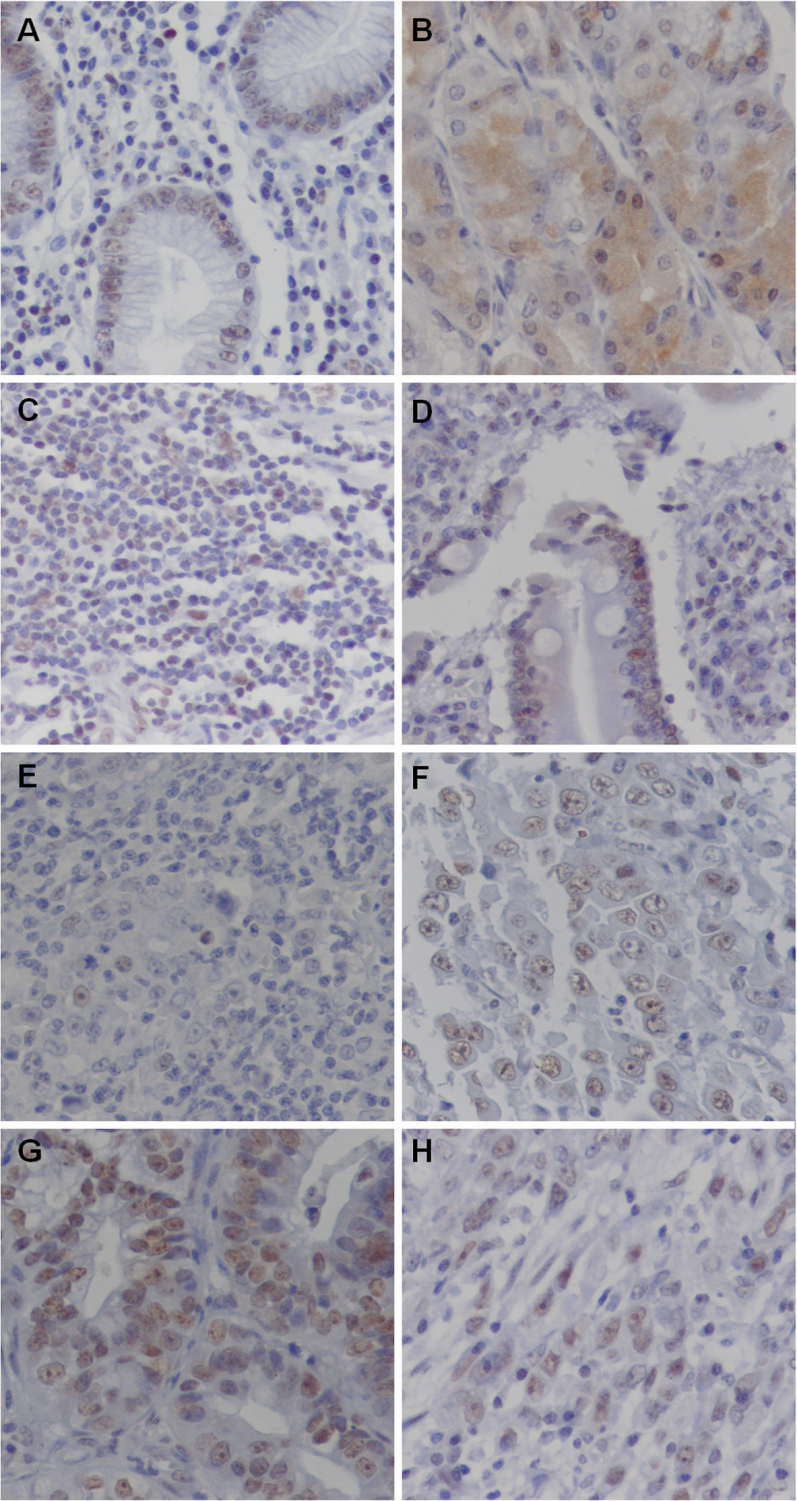
**Immunohistochemical analysis of NPM1 in gastric samples. (A)** NPM1 staining in normal gastric mucosa (400×); **(B)** cytoplasmatic NPM1 staining in parietal cells (400×); **(C)** NPM1 immunoreactivity in inflammatory cells (400×); **(D)** NPM1 staining in intestinal metaplastic cells (400×); **(E)** minimal NPM1 staining in a poorly differentiated tumor (400×); **(F)** weak NPM1 staining in a poorly differentiated tumor (400×); **(G)** moderate NPM1 staining in a moderately differentiated tumor (400×); **(H)** moderate NPM1 staining in a diffuse-type tumor (400×).

In all cases, the NPM1 immunoreactivity was detected in neoplastic and non-neoplastic cells, including in intestinal metaplastic, gastritis and inflammatory cells (Figure 2A-H). NPM1 was mainly expressed in nucleus and nucleolus. Only one case presented cytoplasmatic staining in the parietal cells (Figure 2B). The staining intensity and the percentage of immunoreactive cells varied among the studied cases (Table 2). In nuclei of tumor cells, NPM1 immunoreactivity score ranged from 0 to 2, with 41.7% cases presenting score 0. In nucleoli of tumor cells, 5 of 12 cases (41.7%) presented score 0 and 7 of 12 (58.3%) presented score 2. The score of NPM1 immunoreactivity in the nucleoli of tumor cells was inversely correlated with the protein expression by Western blot (r = -0.693, p = 0.039; by Spearman’s correlation).

**Table 2:** Immunohistochemistry analysis in gastric tumors

**Case**	**Histological subtype**	**Differentiation**	**Stage**	**% of stained tumor cells**	**Location**	**Intensity of staining**	**Score**
1	Intestinal-type	Moderately differentiated	1	60%	Nucleus	Moderate	2
					Nucleolus	Moderate	2
2	Intestinal-type	Poorly differentiated	4	< 10%	Nucleus	Weak	0
					Nucleolus	Moderate	0
3	Intestinal-type	Moderately differentiated	4	40%	Nucleus	Weak	1
					Nucleolus	Moderate	2
4	Intestinal-type	Moderately differentiated	1	20%	Nucleus	Moderate	2
					Nucleolus	Moderate	2
5*	Intestinal-type	Moderately differentiated	1	40%	Nucleus	Weak	1
					Nucleolus	Moderate	2
6	Intestinal-type	Poorly differentiated	3	< 10%	Nucleus	Weak	0
					Nucleolus	Weak	0
7	Intestinal-type	Moderately differentiated	1	< 10%	Nucleus	Weak	0
					Nucleolus	Weak	0
8	Intestinal-type	Moderately differentiated	3	< 10%	Nucleus	Weak	0
					Nucleolus	Weak	0
9	Intestinal-type	Moderately differentiated	4	20%	Nucleus	Weak	1
					Nucleolus	Moderate	2
10	Intestinal-type	Moderately differentiated	2	< 10%	Nucleus	Weak	0
					Nucleolus	Weak	0
11	Intestinal-type	Poorly differentiated	3	40%	Nucleus	Weak	1
					Nucleolus	Moderate	2
12	Diffuse-type	Not applied	3	20%	Nucleus	Moderate	2
					Nucleolus	Moderate	2

*This patient presented strong cytoplasmatic immunoreactivity in normal parietal cells (score = 3).

The *NPM1* mRNA expression did not differ between GC and matched non-neoplastic gastric samples (0.29 ± 0.41 *vs* 0.39 ± 0.42, values relative to a calibrator non-neoplastic sample; *P* = 0.33, by paired t-test; Figure 1D). The *NPM1* mRNA level was reduced at least 1.5-fold in 45.5% of samples and increased in 27.3% of samples (Figure 1E). A moderate inverse correlation was observed between the relative quantifications of NPM1 protein and mRNA levels (r = -0.509; *P* = 0.037, by Pearson’s correlation; Figure 1F).

The intestinal-type GC presented higher *NPM1* mRNA levels than diffuse-type GC (*P* = 0.026, by t-test; Table 1). The mRNA expression was at least 50% reduced in all diffuse-type. In the intestinal-type, the mRNA expression was less than 1.5-fold in 25% of cases and greater than 1.5-fold in 37.5% in relation to their matched non-neoplastic counterpart. On the other hand, the NPM1 protein level did not differ between diffuse-type and intestinal-type GC. However, intestinal-type GC presented a significant reduction of NPM1 protein expression compared to matched non-neoplastic gastric samples (0.94 ± 0.58 *vs* 1.41 ± 0.42, *P* = 0.018, by paired t-test). In addition, the protein level of NPM1 was reduced at least 1.5-fold in 46.2% of intestinal-type GC and in no case of diffuse-type GC.

Tumors from patients with known distant metastasis showed reduced NPM1 protein expression compared to tumors from patients without distant metastasis (*P* < 0.001, by t-test; Table 1). No association between NPM1 expression and any other clinicopathological characteristics was found.

## Discussion

NPM1 is a multifunctional protein. The first proposed role of NPM1 was in the regulation of cell growth, proliferation and transformation because its expression increases in response to mitogenic stimuli and is up-regulated in highly proliferative and malignant cells. However, several recent studies have demonstrated that NPM1 has both proliferative and growth-suppressive roles in the cell (see review [3]).

In the present study, NPM1 protein expression was significantly down-regulated in GC, which supports its role as a tumor suppressor. One NPM1 target is cyclin-dependent kinase inhibitor 2A (CDKNA2) alternate reading frame protein (ARF; also known as p14ARF in humans, and p19Arf in the mouse). ARF protein is involved in cell-cycle arrest and apoptotic processes through inhibition of MDM2 (E3 ubiquitin-protein ligase, a negative regulator of p53) and, therefore, stabilization of p53 [28]. NPM1 acts in the stabilization of ARF within the nucleolus by protecting it from both proteasome-dependent and proteasome-independent degradation. It has been suggested that NPM1 loss of function could lead to an acceleration of tumorigenesis owing to the destabilization and inactivation of ARF, which is known to inhibit cell proliferation through both p53-dependent and p53-independent mechanisms [3,29], in agreement with a potential tumor-suppressor role for NPM1.

The down-regulation of NPM1 was associated with known distant metastasis in patients with GC, suggesting that low levels of NPM1 protein expression may be a marker of poor prognosis in GC if validated in larger clinical study sets. Reduced NPM1 protein level was previously associated with poor outcome in some subtypes of breast cancer [30]. On the other hand, NPM1 overexpression was associated with the presence of distant metastasis in colon cancer [31]. The role of NPM1 may depend on cellular and genetic context. The interaction between NPM1 and MYC may be one of the pathways by which the loss of NPM1 contributes to the development of metastasis. The lack of a functional NPM1 was previously associated with increased levels of MYC [32]. *MYC* is a key oncogene in gastric carcinogenesis (see review [33]), and the overexpression or amplification of the *MYC* locus was previously reported in GC samples and preneoplastic gastric lesions [34-41]. In our population, MYC overexpression was previously associated with the presence of distant metastasis [42]. Moreover, the three tumors of patients with distant metastasis presented MYC immunoreactivity (data not shown).

Here, we observed that NPM1 presented nuclear and nucleolar location. Previous studies showed that NPM1 is a predominantly nucleolar protein, however, a fraction can also be detected in the nucleoplasm (see review [43]). Although the sample size is small, an inverse correlation between nucleoli immunoreactivity and the protein expression (total expression) by Western blot was observed. This finding may be in part to the key role of NPM1 in ribosome biogenesis. In both subcellular compartments, the NPM1 immunoreactivity presented a large inter- and intra-tumor heterogeneity. The NPM1 expression heterogeneity in GC cells may complicate the development of diagnostic tests or treatments targeting the NPM1. Efforts to pharmacologically target NPM1 for cancer therapy might be difficult, due to the fact that its function is likely to be tightly regulated to avoid the possibly detrimental consequences of its decreased or increased function [3].

The NPM1 immunoreactivity was also heterogeneous in intestinal metaplastic, gastritis and inflammatory cells, which are commonly observed in GC patients. NPM1 may also act as an alarmin in the immune system [44]. In macrophages, NPM1 negatively regulates cytokine and chemokine gene expression and their secretion [45]. We hypothesized that the NPM1 expression in tumor cells is modulated in response to microenvironmental stimuli.

We also demonstrated that *NPM1* mRNA expression was inversely correlated with protein expression, which suggests that post-translational mechanisms may be involved in regulating expression of this protein. Previous studies demonstrated that NPM1 protein is modified by ubiquitylation [46-49], which may lead to its depletion despite the elevated mRNA transcription. Proteins make up the cellular machinery and play major roles in most biological processes. Thus, direct assessment of protein levels may often be more informative of the cellular state than analysis of mRNA levels [50]. Protein expression is subject to complex control and is only partly determined by accumulation and degradation of the corresponding mRNAs [51,52]; it is suggested that 20–60% of the variation in steady-state protein abundances is attributable to mRNA levels [53]. It has been speculated that transcriptional bursts, observed to increase variance in mRNA abundance, may be buffered by long protein half-lives [54].

In addition, *NPM1* mRNA expression did not differ between tumors and non-neoplastic samples. Although approximately 45% of tumors presented reduced mRNA expression, about 27% of GC presented more than 1.5-fold increased expression compared to matched non-neoplastic tissue. To our knowledge, only two previous studies evaluated *NPM1* mRNA in gastric tumors by Northern blot. Tanaka et al. [21] reported that 2 of 3 tumors presented hybridization with *NPM1* probe, which was not observed in any of the non-neoplastic samples. You et al. [22] demonstrated that 6 of 7 GC samples presented increased expression compared to non-neoplastic gastric tissue. However, the present study used RT-qPCR, the most sensitive method for detection and quantification of mRNA expression. Additionally, we evaluated a larger number of samples, which may better reflect the heterogeneity of gastric tumors.

Moreover, we observed that intestinal-type GC presented higher mRNA levels than diffuse-type GC, confirming that these two histological GC subtypes follow different genetic pathways and may be two distinct entities [55]. Although *NPM1* mRNA seems to be higher in intestinal-type GC, this subtype showed relatively lower levels of NPM1 protein expression compared to the non-neoplastic samples, which reinforces the inverse correlation between NPM1 protein and mRNA expression.

## Conclusions

We demonstrated that NPM1 down-regulation may have a role in gastric carcinogenesis, especially in intestinal-type GC and in tumors from patients with distant metastasis. However, NPM1 expression presented a large inter- and intra-tumor heterogeneity, which might complicate the development of diagnostic tests or treatments targeting the NPM1. On the other hand, NPM1 protein down-regulation may help in the selection of anticancer treatment strategies based on a better understanding of the pathways deregulated in GC.

## Abbreviations

GC: Gastric cancer; NPM1: Nucleophosmin 1; CHAPS: 3-[(3-cholamidopropyl) dimethylammonio]-1-propanesulfonate; DTT: Dithiothreitol; PVDF: Polyvinylidene difluoride; ACTB: β-Actin; qPCR: quantitative PCR; RT-qPCR: Reverse transcription quantitative PCR; RQ: Relative quantification; SD: Standard deviation; ARF: Cyclin-dependent kinase inhibitor 2A (CDKNA2) alternate reading frame protein; MDM2: E3 ubiquitin-protein ligase.

## Competing interests

The authors declare that they have no competing interests.

## Authors’ contributions

MFL, RC, RRB and MACS conceived and designed the experiments. MFL was involved in literature search, genetic and statistical analysis. TKFM, PDRC and MCM were involved in immunohistochemistry analysis. MFL and DQC were involved in data collection. SD was responsible for pathology analysis. PPA was responsible by samples collection. MFL wrote the first draft of the manuscript. All authors listed have contributed to all subsequent drafts, and have approved the final manuscript.

## Pre-publication history

The pre-publication history for this paper can be accessed here:

http://www.biomedcentral.com/1471-230X/14/9/prepub
